# Food Gels: Fabrication, Characterization, and Application

**DOI:** 10.3390/gels11110886

**Published:** 2025-11-04

**Authors:** Wanwen Chen, Hao Cheng

**Affiliations:** 1 Wuxi Fisheries College, Nanjing Agricultural University, Wuxi 214081, China; chenwanwen@ffrc.cn; 2 Key Laboratory of Integrated Rice-Fish Farming Ecology, Ministry of Agriculture and Rural Affairs, Freshwater Fisheries Research Center, Chinese Academy of Fishery Sciences, Wuxi 214081, China; 3 Sino-US Cooperative International Laboratory for Germplasm Conservation and Utilization of Freshwater Mollusks, Freshwater Fisheries Research Center, Chinese Academy of Fishery Sciences, Wuxi 214081, China; 4 State Key Laboratory of Food Science and Resources, School of Food Science and Technology, Jiangnan University, Wuxi 214122, China

## 1. Introduction

Food gels, typically formulated from proteins, polysaccharides, and lipids, are viscoelastic systems capable of entrapping water (hydrogels), oil (oleogels), and air (aero-gels) within their three-dimensional networks [1]. These gels play a crucial role in modern food formulation and production due to their versatile functional properties, including tailoring the structure of food to achieve a desired appearance, sensory attributes, and textures; incorporating bioactive compounds with high physicochemical stability and bioavailability; enabling the creation of customized food shapes via 3D printing; serving as fat replacers to reduce excessive saturated fatty acid, cholesterol, and calorie intake; and stabilizing metastable food structures and extending their shelf life [1,2,3]. The rational design of food gels can therefore enhance not only food quality and modification, but also its nutritional and health benefits. However, due to their inherent complexity compared to synthetic polymer gels, the relationships between material selection, fabrication strategies, microstructure, and mechanical and functional properties remain insufficiently understood in specific food systems. Moreover, as scientific research often overlooks practical considerations, bridging the gap between theory and application represents a critical challenge in scaling up the production of innovative gel-based foods with enhanced characteristics and functionalities.

This Special Issue comprises recent advancements in food gel technology and their applications across multiple domains, encompassing surimi-based seafood analogs that demand mechanically robust networks and dairy products requiring high emulsification stability and efficient nutrient integration. The innovation of composite gels, which incorporate bioactive components such as enzymatic crosslinkers (e.g., transglutaminase), polysaccharides (e.g., cassia bean gum and soy protein isolate), and nutritional fortificants (e.g., omega-3 fatty acids and iron microcapsules), has further expanded their functional capabilities and health-promoting potential. The research landscape encompasses critical advancements, including low-salt surimi gelation strategies utilizing amino acid supplements and high-pressure processing, emulsion restructuring in milk via homogenization and ultrasonic treatment, and the design of double emulsions (W_1_/O/W_2_) for enhanced nutrient encapsulation. Moreover, it addresses emerging sustainable resource utilization approaches, such as gelatin extraction from underutilized raw materials (e.g., Yanbian cattle bones, and crayfish muscle), biotherapeutic delivery through protein–polysaccharide complexes, and advanced processing technologies like microfluidization and membrane emulsification to improve texture and stability.

The multidisciplinary nature of food gel research requires the integration of protein science, colloidal chemistry, process engineering, and nutritional biology. This interdisciplinary synergy has resulted in significant advances in understanding inter-component interactions, optimizing mechanical properties for targeted food matrices, and developing innovative crosslinking strategies that improve functionality without compromising nutritional quality. Through these developments, food gels continue to pioneer the creation of next-generation food products that support global health and environmental sustainability goals.

## 2. Overview of Papers Published in This Special Issue

This Special Issue, “Food Gels: Fabrication, Characterization, and Application”, brings together nine research articles and two review papers highlighting recent advancements in food gels in terms of their fabrication, characterization, and application. These contributions explore innovative synthesis methods, novel material properties, and diverse applications in products including milk, margarines, Surimi, meat, and bioactive delivery systems.

The study “A Comparative Study of Soy Protein Isolate-κ-Carrageenan Emulsion Gels and Bigels for the Encapsulation, Protection, and Delivery of Curcumin” by Gray et al. examines curcumin delivery performance across soy protein isolate-κ-carrageenan emulsion gels and bigels incorporating glycerol monostearate. The research demonstrates that bigels with optimized glycerol monostearate content significantly enhance curcumin stability and controlled release profiles compared to emulsion gels. Bigels exhibited superior curcumin retention during storage and delayed gastric release while promoting intestinal liberation through improved lipolysis. The findings highlight bigels’ structural advantages for hydrophobic polyphenol delivery in functional food applications.

The study “Improvement of Gel Properties of *Nemipterus virgatus* Myofibrillar Protein Emulsion Gels by Curdlan” by Wu et al. examines the enhancement of emulsion gel properties through the incorporation of curdlan (Cur) into myofibrillar protein (MP) matrices and its application in emulsified surimi. The research demonstrates that Cur, primarily interacting with MP via hydrogen bonding, promotes the formation of a uniform and dense composite network structure, significantly improving adsorption capacity at the oil/water interface. At an optimal concentration of 6% (*w*/*v*), Cur substantially enhances the gel’s hardness, strength, water-holding capacity, and viscoelastic properties. Furthermore, the application of Cur/MP emulsion gels in surimi products outperforms direct oil addition, yielding superior texture, gel strength, and water retention. These findings advance the development of protein–polysaccharide emulsion gels and highlight their potential as functional fat substitutes in seafood products.

The study “Encapsulation of *Lactobacillus reuteri* in Chia-Alginate Hydrogels for Whey-Based Functional Powders” by Cid-Córdoba et al. examines the efficacy of electrohydrodynamic atomization (EHDA) and drip mode (DM) encapsulation techniques for protecting *Lactobacillus reuteri* DSM 17938 within chia mucilage–sodium alginate hydrogels, and their integration into whey-based functional beverage powders. Both EHDA and DM achieved a high encapsulation efficiency (99.0%) and maintained bacterial viability above 9.9 log_10_ CFU/mL post-lyophilization, demonstrating the hydrogel’s exceptional protective capacity. Microstructural analysis revealed well-preserved cell morphology and homogeneous distribution within the polymer matrix, with SEM images confirming spherical, porous microcapsules with surface characteristics influenced by the encapsulation method.

The study “Development, Characterization, and Stability of Margarine Containing Oleogels Based on Olive Oil, Coconut Oil, Starch, and Beeswax” by Naves et al. examines the formulation of margarines using oleogels structured with extra virgin olive oil, coconut oil, corn starch, and beeswax as alternatives to conventional saturated and *trans*-fat-rich spreads. Their key findings reveal that oleogel-based margarines exhibit higher melting temperatures (46–49 °C) and broader melting ranges due to beeswax’s structuring abilities, alongside lower enthalpy values (1.9–2.8 mW) attributed to the higher unsaturated oil content. A color analysis showed a distinct greenish hue from the olive oil, contrasting with commercial samples. Crucially, the oleogel margarines displayed superior thermal stability, resisting oil exudation during thermal cycling, while their commercial counterparts suffered phase separation. The microstructural differences included larger, irregular water droplets in the oleogels, yet their oxidative stability remained acceptable (peroxide values ≤ 9.73 mEq O_2_/kg) owing to olive oil’s natural antioxidants. This research confirms the viability of oleogel-based margarines as functional, healthier options with enhanced thermal performance, oxidative resistance, and reduced saturated fat content.

The paper “Development and Application of Anthocyanin-Based Complex Polysaccharide Gels Based on Blueberry Pomace for Monitoring Beef Freshness” by Zhi et al. examines the fabrication of pH-responsive gels using anthocyanins extracted from blueberry pomace incorporated into chitosan/polyvinyl alcohol (CS/PVA) and starch/PVA (S/PVA) matrices for real-time beef freshness monitoring. The study demonstrated that CS/PVA-BA gels achieved optimal elongation at break (high flexibility), low hydration (8.33% water content), and potent antioxidant activity, while S/PVA-BA gels exhibited superior tensile strength and enhanced pH-sensitive colorimetric responses. Structural analyses confirmed molecular compatibility through hydrogen bonding between anthocyanins and polymer networks. Applied to chilled beef storage at 4 °C, the gels displayed visible color transitions from magenta-red (initial spoilage at 48 h) to bluish-gray (advanced spoilage by day 6), correlating with biochemical spoilage markers (TVB-N > 15 mg/100 g, TVC > 4.0 lg CFU/g). These findings establish a multifunctional platform for intelligent packaging that integrates real-time freshness indication with antioxidant protection, highlighting the potential of waste-derived anthocyanins in sustainable food safety solutions.

The paper “A Mathematical Model of Myosin Heavy Chain Dynamics in the Disintegration of Golden Threadfin Bream *Nemipterus virgatus* Surimi Gel” by Nakamizo et al. investigates the disintegration mechanism of surimi gel in golden threadfin bream, a species characterized by low transglutaminase activity and high protease activity at elevated temperatures. The study focuses on the competition between non-enzymatic polymerization and proteolytic degradation of myosin heavy chain (MHC), a key protein governing gel network formation. Using a kinetic model based on SDS-PAGE analysis of MHC dynamics during heating at 60 °C, the research demonstrates that the model accurately captures MHC depletion, revealing significant degradation of both unpolymerized and polymerized MHC. This degradation directly correlates with the reduced mechanical strength of the gel, highlighting the role of proteolytic activity in gel disintegration. The mathematical framework provides a predictive tool for optimizing heating conditions and controlling the surimi gel’s properties, facilitating the application of underutilized fish species in surimi processing. This work advances the understanding of protein dynamics in food gels and offers practical strategies for improving the quality of seafood products.

The paper “High-Quality Application of Crayfish Muscle in Surimi Gels: Fortification of Blended Gels by Transglutaminase” by Wang et al. investigates the integration of crayfish muscle (0–10%) into silver carp surimi gels and the compensatory role of transglutaminase (TGase) in mitigating structural deterioration. The study demonstrates that crayfish muscle incorporation without TGase progressively reduced the gel’s strength, water-holding capacity (WHC), and structural homogeneity due to disrupted hydrogen bonding, hydrophobic interactions, and disulfide bond formation, as evidenced by decreased whiteness, increased porosity, and free water migration. Conversely, 0.6% TGase addition counteracted these effects by catalyzing ε-(γ-Glu)-Lys crosslinks, enhancing protein aggregation, and promoting shifts in secondary structures from α-helices to β-turns, resulting in denser gel networks, improved WHC, and restored mechanical properties, even at 7.5% crayfish inclusion. TGase-mediated restructuring optimized water distribution and reduced electrostatic repulsion, facilitating disulfide bond formation despite the activity of crayfish-derived proteases. This synergy enables utilization of high-value crayfish byproducts in surimi products, offering a strategy to enhance texture, moisture retention, and sustainability in seafood processing.

The paper “Physicochemical and Functional Properties of Yanbian Cattle Bone Gelatin Extracted Using Acid, Alkaline, and Enzymatic Hydrolysis Methods” by Zhang et al. examines the extraction efficiency, structural integrity, and functional performance of gelatin derived from Yanbian cattle bones using three distinct methods: acid hydrolysis (with hydrochloric acid), alkaline hydrolysis (with sodium hydroxide), and enzymatic hydrolysis (with papain). The study demonstrated that enzymatic hydrolysis with papain achieved the highest yield (25.25%) and optimally preserved collagen’s native structure, resulting in superior hydroxyproline content (19.13 g/100 g), gel strength (259 g), viscosity (521.67 cP), and thermal stability compared to acid and alkaline methods. Structural analyses confirmed that papain extraction minimized protein degradation and maintained the triple-helical conformation, while the amino acid composition revealed enhanced levels of functional residues (e.g., glycine, proline, and hydroxyproline). These findings highlight enzymatic hydrolysis as a mild and efficient approach for producing high-quality, halal-compliant gelatin from underutilized bone byproducts, offering a sustainable alternative for food, pharmaceutical, and biomedical applications.

The paper “Preparation of Cassia Bean Gum/Soy Protein Isolate Composite Matrix Emulsion Gel and Its Effect on the Stability of Meat Sausage” by Zou et al. examines the development and application of emulsion gels formulated with soy protein isolate (SPI) and varying concentrations of cassia bean gum (CG) (0–2%) as fat substitutes in meat sausages. The study demonstrated that emulsion gels with 1.75% CG concentration exhibited optimal structural properties, including the highest gel strength (586.91 g), elasticity (0.94 mm), chewiness (452.94 mJ), water-holding capacity (98.45%), and thermal stability, attributed to enhanced hydrogen bonding and a compact, homogeneous microstructure. When applied as fat replacers in meat sausages, the 1.75% CG/SPI emulsion gel at a 50% substitution level maintained cooking loss, emulsification stability, color, texture, and antioxidant activity comparable to full-fat sausages, while also improving freeze–thaw stability. These findings highlight the potential of CG/SPI emulsion gels as effective fat alternatives for producing low-fat meat products with preserved quality and enhanced functional properties.

The review “Surimi and Low-Salt Surimi Gelation: Key Components to Enhance the Physicochemical Properties of Gels” by Walayat et al. examines strategies to mitigate the textural deterioration of surimi gels in low-salt (reduced NaCl) formulations, focusing on the role of gelation enhancers such as microbial transglutaminase, polyphenols, phosphates, hydrocolloids, amino acids (e.g., *L*-arginine, *L*-lysine), and plant/animal proteins. The study demonstrates that these additives compensate for reduced salt by promoting protein crosslinking, hydrogen bonding, and hydrophobic interactions, thereby improving gel strength, water-holding capacity, and structural integrity. Notably, specific amino acids and protein combinations effectively maintained the properties of the gel comparably to those of full-salt controls, highlighting their potential for producing healthier surimi products without compromising on techno-functional quality. These findings provide a practical framework for the industry to address sodium reduction challenges while meeting consumer demands for nutritious, low-salt seafood alternatives.

The review “Emulsion Structural Remodeling in Milk and Its Gelling Products” by Yao et al. examines the restructuring of milk’s native oil-in-water (O/W) emulsion into three distinct types—restructured single emulsion, mixed emulsion, and double emulsion (W_1_/O/W_2_)—through the incorporation of alternative lipids (e.g., fish oil and flaxseed oil) or pre-formed emulsions, alongside the impact of processing technologies (including heat treatment, high-pressure processing, homogenization, ultrasonic treatment, micro-fluidization, freezing, and membrane emulsification) on microstructure and functional properties. The study demonstrated that these processing methods significantly reduce the dispersed phase size, modify the interfacial layer composition (e.g., enhancing protein adsorption or milk fat globule membrane integrity), and alter the aqueous phase morphology, thereby improving emulsion stability, shelf-life, and sensory characteristics while enabling nutrient encapsulation in double emulsions. This structural remodeling approach facilitates the development of low-fat, nutritionally fortified dairy products with tailored textural and functional attributes, addressing consumer demands for health-focused and sustainable food options.

## 3. Conclusions and Future Perspectives

I would like to acknowledge all the authors who have contributed to this Special Issue, which underscores the significant advancements made in modifying food gels, highlighting their versatility in food applications such as surimi products, meat sausages, dairy items, and gelatin-based materials. The investigations included delve into fundamental gelation mechanisms, advanced processing techniques, and innovative additive strategies to enhance physicochemical properties, reduce salt content, and improve nutritional profiles, all while addressing sustainability and health concerns. Key technological strides include the development of enzymatic hydrolysis methods, like papain extraction from Yanbian cattle bones, which preserved native collagen structures and achieved superior yield, gel strength, viscosity, and thermal stability compared to traditional acid or alkaline approaches. Additionally, the integration of transglutaminase (TGase) and polysaccharides, such as cassia bean gum, fortified gel networks in surimi and meat products, boosting their water-holding capacity, reducing their porosity, and mitigating salt reduction effects through enhanced protein crosslinking. Non-thermal processing technologies, including high-pressure processing, ultrasonication, and microfluidization, optimized emulsion stability in milk-based systems by reducing fat globule size and improving sensory properties without nutrient degradation. Low-salt formulations utilizing amino acids (e.g., *L*-arginine) and hydrocolloids maintained gel integrity and functionality, addressing health risks linked to sodium intake, while the structural remodeling of milk emulsions (single, mixed, and double types) through homogenization and membrane emulsification enabled functional foods with improved nutrient delivery, oxidative stability, and tailored textures for specific consumer needs.

Looking ahead, future research should prioritize scaling up innovative processing techniques, ensuring cost-effectiveness and reproducibility in large-scale food production. This entails developing standardized protocols for evaluating the safety and regulatory compliance of new additives to facilitate faster market entry. Advancing molecular-level understanding of protein–polysaccharide–lipid interactions during gelation will allow for tailored textural customization, particularly for demographic-specific products like those for elderly populations or health-focused diets. Exploring sustainable raw material sources, such as underutilized fish species and agricultural byproducts, can reduce environmental impact and promote circular economy practices. Comprehensive in vivo and clinical studies are needed to validate health benefits like antioxidant or antihypertensive effects, strengthening evidence-based nutritional claims. Leveraging digital technologies, including artificial intelligence and machine learning, will optimize formulation design and predict gel behavior under varying conditions, enabling personalized nutrition solutions. The interdisciplinary collaboration among food scientists, engineers, nutritionists, and regulatory specialists remains crucial for translating laboratory innovations into commercially viable products that meet evolving consumer demands for healthy, sustainable, and high-quality food options, ultimately contributing to global food security and public health.
